# On the Limit Cycles of a Class of Planar Singular Perturbed Differential Equations

**DOI:** 10.1155/2014/379897

**Published:** 2014-03-31

**Authors:** Yuhai Wu, Jingjing Zhou

**Affiliations:** Department of Mathematics, Jiangsu University, Zhenjiang 212013, China

## Abstract

Relaxation oscillations of two-dimensional planar singular perturbed systems with a layer equation exhibiting canard cycles are studied. The canard cycles under consideration contain two turning points and two jump points. We suppose that there exist three parameters permitting generic breaking at both the turning points and the connecting fast orbit. The conditions of one (resp., two, three) relaxation oscillation near the canard cycles are given by studying a map from the space of phase parameters to the space of breaking parameters.

## 1. Introduction

As we know, the second part of Hilbert's 16th Problem is related with the number and distributions of limit cycles of a general polynomial vector field of *n*th degree. Let *H*(*n*) denote the maximum number of limit cycles of a general planar polynomial vector field of degree *n*. As mentioned in [1, 2], there are little studies on an upper bound of *H*(*n*), but there are many results on the lower bounds of *H*(*n*); for example, *H*(3) ≥ 13, *H*(4) ≥ 21, and *H*(5) ≥ 28 (see [3–7] for more details). For the hardness of Hilbert's 16th Problem, according to Smale [8], it might be appropriate to deal with Hilbert's 16th Problem restricted to the classical polynomial Liénard equations. In 2007, Dumortier et al. [9] found 4 limit cycles in a singular perturbed Liénard equation of degree 7 by applying geometric singular perturbation theory, and this result overturns Lin de Melo and Pugh's conjecture (see [9, 10] for details). The general form of planar singular perturbed differential equation can be given by as follows:

(1) $$\begin{array}{c} ɛ\frac{dx}{d\tau }=f\left(x,y,\lambda ,ɛ\right),\\ \frac{dy}{d\tau }=g\left(x,y,\lambda ,ɛ\right), \end{array}$$

where *f*(*x*, *y*, *λ*) and *g*(*x*, *y*, *λ*) are two smooth functions with respect to variables (*x*, *y*, *λ*) ∈ ℝ^2^ × ℝ^*k*^ and *ɛ* is a small real number. As *ɛ* > 0 and small, we make the time scaling *t* = *τ*/*ɛ* and get the following equivalent standard form of slow-fast system which has the same phase portraits as the one of system (1) with the slow variable *x* and the fast variable *y*:

(2) $$\begin{array}{c} \frac{dx}{dt}=f\left(x,y,\lambda \right),\\ \frac{dy}{dt}=ɛg\left(x,y,\lambda \right). \end{array}$$

Let *ɛ* = 0 in system (1) and (2), we, respectively, obtain the following limiting system (3) and (4)

(3) $$\begin{array}{c} f\left(x,y,\lambda \right)=0,\\ \dot{y}=g\left(x,y,\lambda \right), \end{array}$$

(4) x˙=f(x,y,λ),y˙=0.

System (3) and (4) are, respectively, called the reduced equation and the layer equation of system (1). For problems of planar singular perturbed system (1), the reduced equation captures essentially the slow dynamics and the layer equation captures the fast dynamics. The layer equation is a one-dimensional dynamical system in the fast variable *y* with the slow variable *x* acting as a parameter.

The equation *f*(*x*, *y*, *λ*) = 0 defines the critical manifold *S* of the equilibrium of the layer equation (4). The reduced equation describes the dynamics on the critical manifold *S*. Due to geometric singular perturbation theory of Fenichel (see [11] for more details), normally hyperbolic pieces of critical manifolds turn to locally invariant slow manifolds for *ɛ* sufficiently small. Hence under suitable assumptions, orbits of singular perturbed system (2) can be obtained as perturbations of a slow-fast orbit which consist of pieces of the reduced equation (3) and the layer equation (4). Slow-fast orbit is not orbit of system (2), and it is the limit set of system (2) as *ɛ* approaches 0.

At contact points that are isolated points on the critical manifold where normal hyperbolicity is lost, the blow-up method pioneered by Dumortier and Roussarie [12] is a powerful geometric tool in the analysis of orbits of system (2) near nonhyperbolic points. According to the paper [13], we know that the admitted contact points (*x*, *y*, *λ*) = (*x*_0_, *y*_0_, *λ*_0_) of system (2) have been divided into two classes: a generic jump point and a generic turning point. If, after translation, rescaling of the variables (*x*, *y*), and rescaling of time, system (2) can, locally near (0,0, 0), be, respectively, written as

(5) $$\begin{array}{c} \dot{x}=y-x^{2}+x^{3}h_{1}\left(x,y,ɛ,\lambda \right),\\ \dot{y}=-ɛ\left(1+xh_{2}\left(x,y,ɛ,\lambda \right)\right), \end{array}$$

or

(6) $$\begin{array}{c} \dot{x}=y-x^{2}+x^{3}h_{1}\left(x,y,ɛ,\lambda \right),\\ \dot{y}=-ɛ\left(a\left(\lambda \right)+x+x^{2}h_{2}\left(x,y,ɛ,\lambda \right)\right), \end{array}$$

then correspondingly nonhyperbolic point (*x*_0_, *y*_0_, *λ*_0_) of system (2) is called a generic jump point or a generic turning point of system (2), where functions *h*_1_ and *h*_2_ are smooth and *a*(*λ*_0_) = 0 and *a*(*λ*) is smooth at *λ*_0_ in case of a generic turning point.

**Figure 1:**
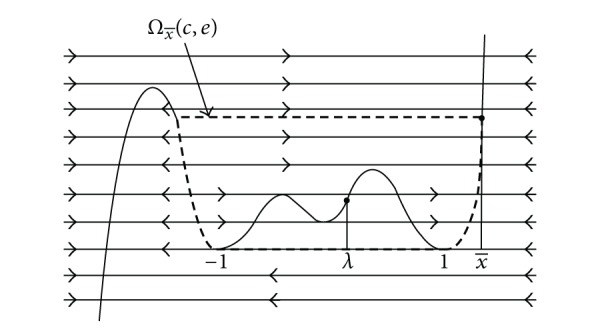
Canard cycle having two jump points.

If a slow-fast orbit of system (2) denoted by Γ is closed, then Γ is called slow-fast cycle. Further a slow-fast cycle Γ is called common (see [9]) if all its slow curves have the same type: attracting or repelling. A slow-fast cycle Γ is called a canard cycle if it contains both attracting and repelling slow curves. Here slow-fast cycle is not periodic orbit of system (2), but a limit periodic set as *ɛ* approaches 0 and the limit cycles that are close to slow-fast cycle are called relaxation oscillations. In 2007, Dumortier et al. [9] proved that at least three limit cycles of Liénard equations with well-chosen polynomial *f* of degree 7 bifurcate from the canard cycle consisting of two jump points by analyzing the zeros of slow divergence integral of canard cycle (see Figure 1).

**Figure 2:**
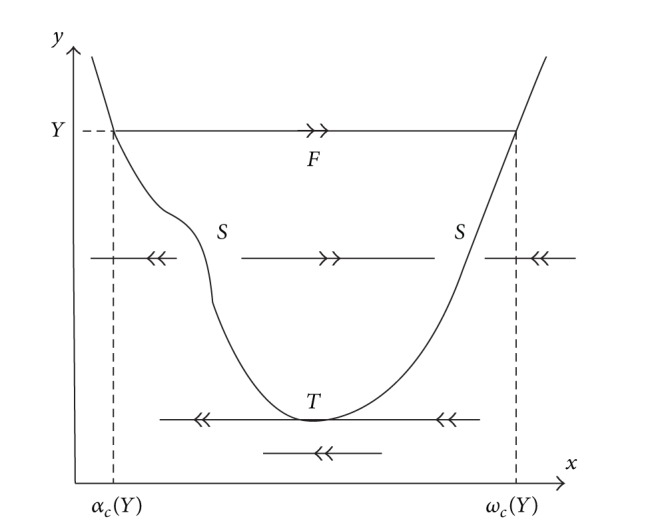
Canard cycle having one turning point.

In 2011, de Maesschalck and Dumortier [10] showed that four limit cycles bifurcated from the canard cycle of a singular perturbed Liénard system of degree six, which consists of a single fast orbit and a single slow curve and contains one turning point (see Figure 2). It can be clearly checked that the slow curve on one side of the turning point is attracting, and the part on the other side is repelling. This kind of canard cycle is said to be a 1-breaking parameter family of canard cycle. The main method used is to study the zeros of slow divergence integral of canard cycle.

**Figure 3:**
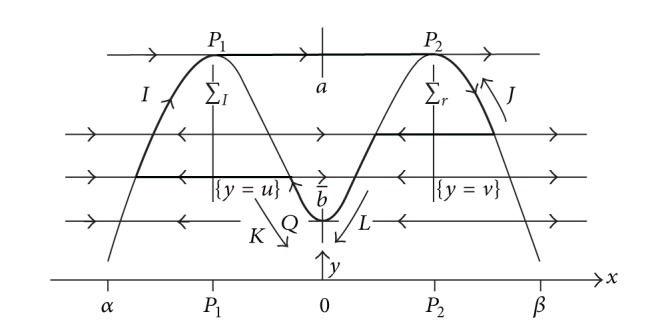
Canard cycles having two jump points and one turning point.

In 2007, Dumortier and Roussarie [14, 15] considered two-dimensional slow-fast systems with a layer equation exhibiting canard cycle which contains a turning point and a fast orbit connecting two jump points (see Figure 3). At both the turning point and jump point, the presence of two parameters permitting generic breaking is assumed. The conditions of existing one (two or three) limit cycle in the above planar system are given by studying the fixed points of the Poincare map near canard cycles.

**Figure 4:**
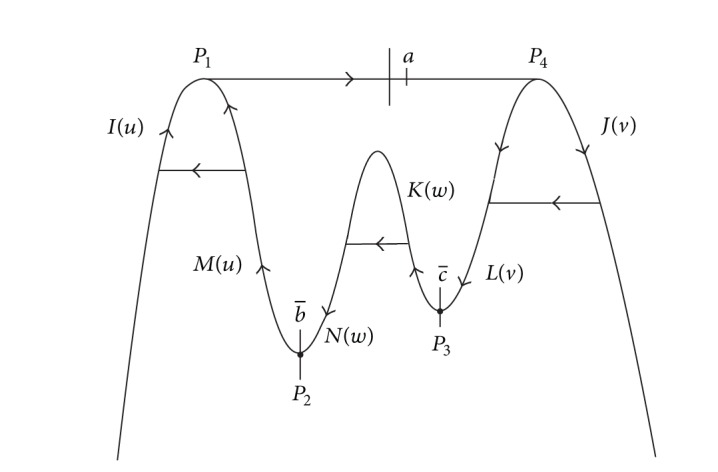
Canard cycle having two jump points and two turning points.

In this paper, we want to study the number of limit cycles near canard cycle which contains two turning points *p*_2_, *p*_3_ and two jump points *p*_1_, *p*_4_ that allow three breaking mechanisms and each one corresponds to one phase parameter (see Figure 4).

This paper is organized as follows. Our main results will be presented in the first part. The proofs of the results are presented in the second part. In the third part, a concrete example of planar singular Liénard equation existing three limit cycles will be given.

Consider the following smooth slow-fast Liénard system *X*_*λ*_,_*ɛ*_:

(7) $$\begin{array}{c} X_{\lambda ,ɛ}:\{\begin{array}{c} \dot{x}=y-F_{a}\left(x\right),\\ \dot{y}=ɛg\left(x,b,c\right), \end{array}\;\lambda =\left(a,b,c\right)\in {\mathbb{R}}^{3}, \end{array}$$

where *λ* = (*a*, *b*, *c*) is near (0,0, 0).

In the following, we assume that the smooth functions *F*_*a*_(*x*), *g*(*x*, *b*, *c*) fulfill the following conditions.

(*H* _1_) On the interval (−*x*_0_, *x*_0_), as *a* = 0, the function *F*_0_(*x*) has five singular points: two Morse maximum points at *p*_1_, *p*_4_, two Morse minimum points at *p*_2_, *p*_3_, and one Morse maximum at *p*_0_ with −*x*_0_ < *p*_1_ < *p*_2_ < *p*_0_ < *p*_3_ < *p*_4_ < *x*_0_. The parameter *a* is just the difference *a* = *F*_*a*_(*p*_4_) − *F*_*a*_(*p*_1_). Let *α*_0_ = *F*_0_(0), *α*_1_ = *F*_0_(*p*_2_), *α*_2_ = *F*_0_(*p*_3_), *α* = max⁡{*α*_1_, *α*_2_}, and *β* = *F*_0_(*p*_4_) = *F*_0_(*p*_1_). Then the values *F*_0_(±*x*_0_) are assumed to be below the minimum value min⁡{*α*_1_, *α*_2_}.
(*H* _2_) Suppose that *g*(0,0, 0) = 0, *g*(0,0, *p*_2_) = 0, *g*(0,0, *p*_3_) = 0, *g*(0,0, *p*_1_) ≠ 0, and *g*(0,0, *p*_4_) ≠ 0, but ∂*g*(0,0, *p*_*i*_)/∂*x* ≠ 0, *i* = 2,3, ∂*g*(0,0, *p*_2_)/∂*b* ≠ 0 and ∂*g*(0,0, *p*_3_)/∂*c* ≠ 0, *g*(0,0, *x*) > 0 for *p*_1_ < *x* < *p*_2_ or *p*_0_ < *x* < *p*_3_, *g*(0,0, *x*) < 0 for *p*_2_ < *x* < *p*_0_ or *p*_3_ < *x* < *p*_4_ (see Figure 4).
(*H* _3_) When *λ* = (0,0, 0), there exists a canard cycle containing four horizontal segments: one between the two Morse maxima *x* = *p*_1_, *x* = *p*_4_ denoted by *σ*_*h*_, one below the left Morse maximum value and at the height *y* = *u* denoted by *σ*_*l*_, one below the right Morse maximum value and at the height *y* = *v* denoted by *σ*_*r*_(*v*), and one at the height *y* = *w* and between *p*_2_ and *p*_3_ denoted by *σ*_*m*_, where *u* ∈ (*α*_1_, *β*), *v* ∈ (*α*_2_, *β*), *w* ∈ (*α*, *α*_0_), and the corresponding canard cycle is denoted by Γ_*uvw*_.

An essential tool to study the limit cycles bifurcated from the canard cycle is the slow divergence integral (see [9, 10, 13–15]); the slow divergence integral of the slow curve of system (7) between *x*_1_, *x*_2_ is defined as follows:

(8) $$\begin{array}{c} \text{Int}\left(x_{1},x_{2}\right)=-\overset{x_{2}}{\underset{x_{1}}{\int }}\frac{1}{g\left(x,0,0\right)}{\left(\frac{dF_{0}\left(x\right)}{dx}\right)}^{2}dx‍. \end{array}$$

Consider canard cycle Γ_*uvw*_ of system (7). Let *x*_1_(*u*), *x*_2_(*u*), respectively, denote *x*-coordinates of intersection points between *σ*_*l*_ and slow curve *y* = *F*_0_(*x*), where −*x*_0_ < *x*_2_(*u*) < *x*_1_(*u*) < *p*_2_; let *x*_1_(*w*), *x*_2_(*w*), respectively, denote *x*-coordinates of intersection points between *σ*_*m*_ and *y* = *F*_0_(*x*), where *p*_2_ < *x*_1_(*w*) < *x*_2_(*w*) < *p*_3_; let *x*_1_(*v*), *x*_2_(*v*), respectively, denote *x*-coordinates of intersection points between *σ*_*r*_ and curve *y* = *F*_0_(*x*), where *p*_3_ < *x*_1_(*v*) < *x*_2_(*v*) < *x*_0_. By applying the slow divergence integral formula introduced in (8) to canard cycle Γ_*uvw*_ of system (7), we get the following six integrals:

(9) $$\begin{array}{c} I\left(u\right)=\text{Int}\left(x_{2}\left(u\right),p_{1}\right),\;\;M\left(u\right)=\text{Int}\left(x_{1}\left(u\right),p_{2}\right),\\ N\left(w\right)=\text{Int}\left(x_{1}\left(w\right),p_{2}\right),\;\;K\left(w\right)=\text{Int}\left(x_{2}\left(w\right),p_{3}\right),\\ L\left(v\right)=\text{Int}\left(x_{1}\left(v\right),p_{3}\right),\;\;J\left(v\right)=\text{Int}\left(x_{2}\left(v\right),p_{4}\right). \end{array}$$

It is easy to check that

(10) M′(u),L′(v),N′(w),K′(w)<0,I′(u),J′(v)>0, u∈(α1,β),v∈(α2,β), w∈(α,α0).

Then, the canard cycle Γ_*uvw*_ is associated with the above six functions: *I*(*u*), *J*(*v*), *K*(*w*), *L*(*v*), *M*(*u*), and *N*(*w*), which are the slow divergence integrals of six slow curves contained in Γ_*uvw*_. In detail, two of these curves are located on the left of *p*_2_ and their slow divergence integrals are functions of *u*, two of them are on the right of *p*_3_ and their slow divergence integrals are functions of *v*, and two of them are between *p*_2_ and *p*_3_ and their slow divergence integrals are functions of *w* (see Figure 4).

Now we give the following main results.

**Theorem 1**

Consider a general slow-fast Liénard system (7). Suppose that the smooth functions *F*_*a*_(*x*), *g*(*x*, *b*, *c*) fulfill the conditions *H*_1_, *H*_2_, *H*_3_. Let *D*(*u*, *v*, *w*) = *J*(*v*) − *I*(*u*) + *M*(*u*) − *N*(*w*) + *K*(*w*) − *L*(*v*) denote the total slow divergence integral of Γ_*uvw*_ of system (7).

1. If there exist *u*_0_ ∈ (*α*_1_, *β*), *v*_0_ ∈ (*α*_2_, *β*), and *w*_0_ ∈ (*α*, *α*_0_) such that *D*(*u*_0_, *v*_0_, *w*_0_) ≠ 0, then for *ɛ* > 0 and small enough (*u*_0_, *v*_0_, *w*_0_) is a regular point of Φ_*ɛ*_, whose explicit expression will be given in Section 2 and system (7) has a hyperbolic relaxation oscillation which is near Γ_*uvw*_.
2. If there exist *u*_0_ ∈ (*α*_1_, *β*), *v*_0_ ∈ (*α*_2_, *β*), and *w*_0_ ∈ (*α*, *α*_0_) such that *I*(*u*_0_) − *J*(*v*_0_) > 0, *M*(*u*_0_) − *N*(*w*_0_) = 0, and *I*(*u*_0_) − *J*(*v*_0_) = *K*(*w*_0_) − *L*(*v*_0_), then for *ɛ* > 0 and small enough there exits (*u*_0_(*ɛ*), *v*_0_(*ɛ*), *w*_0_(*ɛ*)) that is a generic fold singularity of Φ_*ɛ*_. A relaxation oscillation bifurcates from Γ_*uvw*_ and this semistable limit cycle is generically unfolded by the parameter (*a*, *b*, *c*) for *ɛ* > 0 and small enough, producing a pair of hyperbolic limit cycles of system (7).
3. If there exist *u*_0_ ∈ (*α*_1_, *β*), *v*_0_ ∈ (*α*_2_, *β*), and *w*_0_ ∈ (*α*, *α*_0_) such that *J*(*v*_0_) − *I*(*u*_0_) = 0, *M*(*u*_0_) − *N*(*w*_0_) = 0, *L*(*v*_0_) − *K*(*w*_0_) = 0, and *J*′(*v*_0_)*M*′(*u*_0_)*K*′(*w*_0_) − *I*′(*u*_0_)*L*′(*v*_0_)*N*′(*w*_0_) ≠ 0, then for *ɛ* > 0 and small enough there exits $\left({\hat{u}}_{0}(ɛ\right)$ , ${\hat{v}}_{0}(ɛ)$ , ${\hat{w}}_{0}(ɛ))$ that is a generic cusp singularity of Φ_*ɛ*_. A codimension 2 relaxation oscillation bifurcates from Γ_*uvw*_ and this degenerated limit cycle is generically unfolded by the parameter (*a*, *b*, *c*) for *ɛ* > 0 and small enough, producing system (7) having three hyperbolic limit cycles in the vicinity of canard cycle Γ_*uvw*_.

## 2. The Proof of Main Results

To study the limit cycle of the *X*_*λ*,*ɛ*_ near the canard cycle Γ_*uvw*_, we choose one vertical section *C*_1_ at *x* = 0, cutting the segment *σ*_*h*_, section *C*_2_ transversal to the turning point *p*_2_, and section *C*_3_ transversal to the turning point *p*_3_. The parameter *a* is the breaking parameter for the section *C*_1_, a rescaling of *b*, given by $\bar{b}=\varepsilon^{-1/2}b$ , is the breaking parameter at *C*_2_, and a rescaling of *c*, given by $\bar{c}=\varepsilon^{-1/2}c$ , is the breaking parameter at *C*_3_ (see [13] for more details). In the following, we denote $\left(a,\bar{b},\bar{c}\right)$ by $\bar{\lambda }$ . Let ∑_*l*_, ∑_*m*_, and ∑_*r*_ be three sections which are transverse to the horizontal segments *σ*_*l*_, *σ*_*m*_, *σ*_*r*_, parameterized, respectively, by *u*, *v*, *w*.

To study the fixed points of the obtained Poincaré map, first we give the following definition of *ɛ*-regularly smooth function and the following lemma by introducing the relationship between intermediate variables *u*, *v*, *w* and $a,\bar{b},\bar{c}$ .

**Definition 2 (see [9, 13])**

A function *f*(*z*, *ɛ*), with *z* ∈ ℝ^*p*^ for some *p* ∈ *ℕ*, is called *ɛ*-regularly smooth in *z*, if *f* is continuous and all partial derivatives of *f* with respect to *z* exist and are continuous in (*z*, *ɛ*).

**Lemma 3**

For *ɛ* > 0 small enough, a limit cycle of system (7) cuts ∑_*l*_ in *u*, ∑_*m*_ in *w*, and ∑_*r*_ in *v* if and only if $\left(a,\bar{b},\bar{c})=\Phi_{ɛ}(u,v,w\right)$ , where Φ_*ɛ*_ is given by the following:

(11) $$\begin{array}{c} \begin{array}{c} \Phi_{ɛ}:\{\begin{array}{cc} a=\exp\left(\frac{\bar{I}\left(u,ɛ\right)}{ɛ}\right)-\exp\left(\frac{\bar{J}\left(v,ɛ\right)}{ɛ}\right), & \\ \bar{b}=\exp\left(\frac{\bar{M}\left(u,ɛ\right)}{ɛ}\right)-\exp\left(\frac{\bar{N}\left(w,ɛ\right)}{ɛ}\right), & \\ \bar{c}=\exp\left(\frac{\bar{K}\left(w,ɛ\right)}{ɛ}\right)-\exp\left(\frac{\bar{L}\left(v,ɛ\right)}{ɛ}\right), & \end{array} \end{array} \end{array}$$

where $\bar{I}(u,ɛ)$ , $\bar{J}(v,ɛ)$ , $\bar{K}(w,ɛ)$ , $\bar{L}(v,ɛ)$ , $\bar{M}(u,ɛ)$ , and $\bar{N}(w,ɛ)$ are *ɛ*-regularly smooth in *u*, *v*, *w*, and, respectively, equal to *I*(*u*), *J*(*v*), *K*(*w*), *L*(*v*), *M*(*u*), and *N*(*w*) for *ɛ* = 0.

**Proof**

From [13–15], due to the chosen orientation on the *C*_*i*_  (*i* = 1,2, 3), the transitions have the following expressions:

1. from ∑_*l*_ to *C*_1_: $u\to \exp(\bar{I}(u,\bar{\lambda },ɛ)/ɛ)+f_{l}(\bar{\lambda },ɛ)$ ,
2. from ∑_*l*_ to *C*_2_: $u\to \exp(\bar{M}(u,\bar{\lambda },ɛ)/ɛ)+g_{l}(\bar{\lambda },ɛ)$ ,
3. from ∑_*m*_ to *C*_2_: $w\to \exp(\bar{N}(w,\bar{\lambda },ɛ)/ɛ)+h_{m_{1}}(\bar{\lambda },ɛ)$ ,
4. from ∑_*m*_ to *C*_3_: $w\to \exp(\bar{K}(w,\bar{\lambda },ɛ)/ɛ)+h_{m_{2}}(\bar{\lambda },ɛ)$ ,
5. from ∑_*r*_ to *C*_1_: $v\to \exp(\bar{J}(v,\bar{\lambda },ɛ)/ɛ)+f_{r}(\bar{\lambda },ɛ)$ ,
6. from ∑_*r*_ to *C*_3_: $v\to \exp(\bar{L}(v,\bar{\lambda },ɛ)/ɛ)+g_{r}(\bar{\lambda },ɛ)$ ,

where functions *f*_*l*_, *g*_*l*_, *f*_*r*_, *g*_*r*_, *h*_*m*_1__, *h*_*m*_2__ are *ɛ*-regularly smooth in $\bar{\lambda }$ , and functions $\bar{I}(u,\bar{\lambda },ɛ)$ , $\bar{K}(w,\bar{\lambda },ɛ)$ , $\bar{L}(v,\bar{\lambda },ɛ)$ , $\bar{M}(u,\bar{\lambda },ɛ)$ , $\bar{N}(w,\bar{\lambda },ɛ)$ , $\bar{J}(v,\bar{\lambda },ɛ)$ are *ɛ*-regularly smooth in $\bar{\lambda }$ and satisfy that $\bar{I}(u,0,0)=I(u)$ , $\bar{K}(w,0,0)=K(w)$ , $\bar{L}(v,0,0)=L(v)$ , $\bar{M}(u,0,0)=M(u)$ , $\bar{J}(v,0,0)=J(v)$ , and $\bar{N}(w,0,0)=N(w)$ .

By using the same analysis as [14, 15], we get that the system of equations for the existence of limit cycles of system (7) is

(12) exp⁡(I¯(u,λ¯,ɛ)ɛ)−exp⁡(J¯(v,λ¯,ɛ)ɛ)  =fr(λ¯,ɛ)−fl(λ¯,ɛ)=aF(λ¯,ɛ),exp⁡(M¯(u,λ¯,ɛ)ɛ)−exp⁡(N¯(w,λ¯,ɛ)ɛ)  =hm1(λ¯,ɛ)−gl(λ¯,ɛ)=b¯H(λ¯,ɛ),exp⁡(K¯(w,λ¯,ɛ)ɛ)−exp⁡(L¯(v,λ¯,ɛ)ɛ)  =gr(λ¯,ɛ)−hm2(λ¯,ɛ)=c¯G(λ¯,ɛ),

where $F(\bar{\lambda },0)\neq 0$ , $G(\bar{\lambda },0)\neq 0$ , and $H(\bar{\lambda },0)\neq 0$ . Rewrite the above equations into the following form:

(13) $$\begin{array}{c} \exp\left(\frac{\bar{I}\left(u,\bar{\lambda },ɛ\right)}{ɛ}\right)-\exp\left(\frac{\bar{J}\left(v,\bar{\lambda },ɛ\right)}{ɛ}\right)=a,\\ \exp\left(\frac{\bar{M}\left(u,\bar{\lambda },ɛ\right)}{ɛ}\right)-\exp\left(\frac{\bar{N}\left(w,\bar{\lambda },ɛ\right)}{ɛ}\right)=\bar{b},\\ \exp\left(\frac{\bar{K}\left(w,\bar{\lambda },ɛ\right)}{ɛ}\right)-\exp\left(\frac{\bar{L}\left(v,\bar{\lambda },ɛ\right)}{ɛ}\right)=\bar{c}, \end{array}$$

where new functions $\overset{-}{I}$ , $\overset{-}{J}$ , $\overset{-}{K}$ , $\overset{-}{L}$ , $\overset{-}{M}$ , and $\overset{-}{N}$ differ from the previous ones in terms of order *o*(*ε*) and are *ε*-regularly smooth in $\left(u,v,w,\overset{-}{\lambda }\right)$ .

We can solve this system in *a*, $\bar{b}$ , and $\bar{c}$ because the partial derivations of the left hand term with respect to *a*, $\bar{b}$ , and $\bar{c}$ are flat in *ɛ*. So one can solve (13) to obtain *a* = *a*(*u*, *v*, *w*, *ɛ*), $\bar{b}=b(u,v,w,ɛ)$ , and $\bar{c}=c(u,v,w,ɛ)$ , such that the functions *a*(*u*, *v*, *w*, *ɛ*), $\bar{b}(u,v,w,ɛ)$ , and $\bar{c}(u,v,w,ɛ)$ have the same form as the left hand term of (13) except that the functions $\bar{I}$ , $\bar{J}$ , $\bar{K}$ , $\bar{L}$ , $\bar{M}$ , $\bar{N}$ are replaced by new functions not depending on $\bar{\lambda }$ and which are *ɛ*-flat perturbations of the previous ones. We will continue to call them $\bar{I}(u,ɛ)$ , $\bar{J}(v,ɛ)$ , $\bar{K}(w,ɛ)$ , $\bar{L}(v,ɛ)$ , $\bar{M}(u,ɛ)$ , $\bar{N}(w,ɛ)$ , and $\bar{I}(u,0)=I(u)$ , $\bar{J}(v,0)=J(v)$ , $\bar{K}(w,0)=K(w)$ , $\bar{L}(v,0)=L(v)$ , $\bar{M}(u,0)=M(u)$ , $\bar{N}(w,0)=N(w)$ .

The proof of Lemma 3 is completed.

### 2.1. The Proof of the First Part of Theorem 1

We take *ɛ* small enough and we view Φ_*ɛ*_ as a map from (*u*, *v*, *w*) to $\left(a,\bar{b},\bar{c}\right)$ . First by direct computation, we get the Jacobian matrix of map Φ_*ɛ*_ as follows:

(14) $$\begin{array}{c} \frac{\partial \Phi_{ɛ}\left(u,v,w\right)}{\partial \left(u,v,w\right)}=\left(\begin{array}{ccc} \frac{{\bar{I}}^{\prime }e^{\bar{I}/ɛ}}{ɛ} & -\frac{{\bar{J}}^{\prime }e^{\bar{J}/ɛ}}{ɛ} & 0\\ \frac{{\bar{M}}^{\prime }e^{\bar{M}/ɛ}}{ɛ} & 0 & -\frac{{\bar{N}}^{\prime }e^{\bar{N}/ɛ}}{ɛ}\\ 0 & -\frac{{\bar{L}}^{\prime }e^{\bar{L}/ɛ}}{ɛ} & \frac{{\bar{K}}^{\prime }e^{\bar{K}/ɛ}}{ɛ} \end{array}\right), \end{array}$$

where ${\bar{I}}^{\prime }$ , ${\bar{M}}^{\prime }$ are the partial derivatives with respect to *u*, ${\bar{J}}^{\prime }$ , ${\bar{L}}^{\prime }$ are the partial derivatives with respect to *v*, and ${\bar{K}}^{\prime }$ , ${\bar{M}}^{\prime }$ are the partial derivatives with respect to *w*.

Let Δ(*u*, *v*, *w*, *ɛ*) = det⁡(∂Φ_*ɛ*_(*u*, *v*, *w*)/∂(*u*, *v*, *w*)). To find the singular points of map Φ_*ɛ*_, by direct computation we get the following formula about the determinant Δ(*u*, *v*, *w*, *ɛ*):

(15) $$\begin{array}{c} \Delta =\frac{1}{\varepsilon^{3}}\left(e^{\left(\bar{J}+\bar{K}+\bar{M}\right)/ɛ}{\bar{J}}^{\prime }{\bar{K}}^{\prime }{\bar{M}}^{\prime }-e^{\left(\bar{I}+\bar{L}+\bar{N}\right)/ɛ}{\bar{I}}^{\prime }{\bar{L}}^{\prime }{\bar{N}}^{\prime }\right). \end{array}$$

Let $\bar{D}=\bar{J}-\bar{I}+\bar{K}-\bar{L}+\bar{M}-\bar{N}$ ; then $D(u,v,w)=\bar{D}(u,v,w,0)$ is exactly the complete slow divergence integral computed along the canard cycle Γ_*uvw*_. With the function $\bar{D}$ , we can rewrite Δ into

(16) $$\begin{array}{c} \Delta =\frac{1}{\varepsilon^{3}}{\bar{J}}^{\prime }{\bar{K}}^{\prime }{\bar{M}}^{\prime }e^{\left(\bar{I}+\bar{L}+\bar{N}\right)/ɛ}\left(e^{\bar{D}/ɛ}-\frac{{\bar{I}}^{\prime }{\bar{L}}^{\prime }{\bar{N}}^{\prime }}{{\bar{J}}^{\prime }{\bar{K}}^{\prime }{\bar{M}}^{\prime }}\right). \end{array}$$

For *ɛ* > 0 and small, the equation for the singular points of the map Φ_*ɛ*_ given by Δ(*u*, *v*, *w*, *ɛ*) = 0 is equivalent to the equation

(17) $$\begin{array}{c} E\left(u,v,w,ɛ\right)=\bar{D}\left(u,v,w,ɛ\right)-ɛ\log\frac{{\bar{I}}^{\prime }{\bar{L}}^{\prime }{\bar{N}}^{\prime }}{{\bar{J}}^{\prime }{\bar{K}}^{\prime }{\bar{M}}^{\prime }}=0. \end{array}$$

It follows from (10) that the term in the logarithm in (17) is strictly positive. Under the conditions of the first part of Theorem 1, we can get that $\bar{D}\neq 0$ as *ɛ* > 0 and small enough by noting *ɛ*-regularity of function $\bar{D}(u,v,w,ɛ)$ . That means that the map Φ_*ɛ*_ is nondegenerated at the point (*u*_0_, *v*_0_, *w*_0_), so from Lemma 3, we get that system (7) has one limit cycle near Γ_*u*_0_ *v*_0_ *w*_0__.

So the conclusion of the first part of Theorem 1 follows.

### 2.2. The Proof of the Second Part of Theorem 1

In this subsection, we give the proof of the second part of Theorem 1.

First, we present the following lemma.

**Lemma 4**

As *ɛ* > 0 and small enough, if there exists *P*_0_(*ɛ*) = (*u*_0_(*ɛ*), *v*_0_(*ɛ*), *w*_0_(*ɛ*)) that satisfies Δ(*u*, *v*, *w*, *ɛ*) = 0, then it holds that

(18) $$\begin{array}{c} grad\;\Delta {|}_{P_{0}(ɛ)}=k\cdot grad\;E{|}_{P_{0}(ɛ)}, \end{array}$$

where *grad* Δ = (∂Δ/∂*u*, ∂Δ/∂*v*, ∂Δ/∂*w*) is the gradient of function Δ(*u*, *v*, *w*, *ɛ*) and *k* is nonzero function at (*P*_0_(*ɛ*), *ɛ*).

**Proof**

From (10) in Section 1, we get that equation *E*(*u*, *v*, *w*, *ɛ*) = 0 determines a surface *S*_1_ in the neighborhood of point *P*_0_(*u*_0_, *v*_0_, *w*_0_) for *ɛ* > 0 and small. From the fact that Δ(*u*, *v*, *w*, *ɛ*) = 0 is equivalent to *E*(*u*, *v*, *w*, *ɛ*) = 0, we get that the equation Δ(*u*, *v*, *w*, *ɛ*) = 0 also determines the surface *S*_1_. For grad Δ|_*P*_0_(*ɛ*)_, grad *E*|_*P*_0_(*ɛ*)_ are both the normal vectors of surface *S*_1_ at the point *P*_0_(*ɛ*), then we get (18).

The proof of Lemma 4 is completed.

*Proof of the Second Part of Theorem 1*. First, we introduce functions *φ*(*u*, *v*, *ɛ*), *ψ*(*u*, *v*, *ɛ*), *ϕ*(*u*, *v*, *ɛ*) as follows:

(19) $$\begin{array}{c} \Phi_{ɛ}:\{\begin{array}{cc} a=\exp\left(\frac{\bar{I}\left(u,ɛ\right)}{ɛ}\right)-\exp\left(\frac{\bar{J}\left(v,ɛ\right)}{ɛ}\right) & \\ \;\equiv \varphi \left(u,v,ɛ\right), & \\ \bar{b}=\exp\left(\frac{\bar{M}\left(u,ɛ\right)}{ɛ}\right)-\exp\left(\frac{\bar{N}\left(w,ɛ\right)}{ɛ}\right) & \\ \;\equiv \psi \left(u,w,ɛ\right), & \\ \bar{c}=\exp\left(\frac{\bar{K}\left(w,ɛ\right)}{ɛ}\right)-\exp\left(\frac{\bar{L}\left(v,ɛ\right)}{ɛ}\right) & \\ \;\equiv \phi \left(v,w,ɛ\right), & \end{array} \end{array}$$

where the map Φ_*ɛ*_ is given in Lemma 3.

Denote (*u*_0_, *v*_0_, *w*_0_) by *P*_0_, from assumptions in the second part of Theorem 1; then we get *E*(*P*_0_, 0) = 0 and (∂*E*/∂*u* · ∂*E*/∂*v*)|_(*P*_0_,0)_ ≠ 0. For *ɛ* > 0 and small, by applying Implicit Function Theorem, we can find surface $\tilde{S}:v=v(u,w,ɛ)$ which satisfies the equation *E*(*u*, *v*, *w*, *ɛ*) = 0. On surface $\tilde{S}$ , we choose point (*u*_0_(*ɛ*), *v*_0_(*ɛ*), *w*_0_(*ɛ*)) denoted by *P*_0_(*ɛ*) such that *E*(*P*_0_(*ɛ*), *ɛ*) = 0, *P*_0_(0) = *P*_0_; that is, Δ(*P*_0_(*ɛ*), *ɛ*) = 0.

From (∂*φ*/∂*v*)|_(*P*_0_,0)_ ≠ 0 and Implicit Function Theorem, we can get *v* = *v*(*a*, *u*, *ɛ*) from the first equation of (19). Also, from (∂*ϕ*/∂*w*)|_(*P*_0_,0)_ ≠ 0, we can get $w=w(\bar{c},v,ɛ)$ from the third one. Substituting *v* = *v*(*a*, *u*, *ɛ*), $w=w(\bar{c},v,ɛ)$ into the second one, we get $\bar{b}=\psi (u,w(\bar{c},v,ɛ),ɛ)\equiv \bar{\psi }(u,a,\bar{c},ɛ)$ .

Next, we compute the first derivative of $\bar{b}$ with respect to *u* as

(20) db¯du=ψu+ψwdwdu=ψu+ψw∂w∂v∂v∂u=ψu+ψw(−ϕvϕw)(−φuφv)=−Δϕwφv.

Then, as Δ(*u*, *v*, *w*, *ɛ*) = 0 we compute the second derivative of $\bar{b}$ with respect to *u* as

(21) $$\begin{array}{c} {\frac{d^{2}\bar{b}}{du^{2}}|}_{\Delta =0}=\frac{d}{du}{\left(\frac{-\Delta }{\phi_{w}\varphi_{v}}\right)|}_{\Delta =0}=-\frac{d\Delta /du}{\phi_{w}\varphi_{v}}. \end{array}$$

From (10), we get that (∂*D*/∂*u*)|_*P*_0__ = *M*′(*u*_0_) − *I*′(*u*_0_) < 0, (∂*D*/∂*v*)|_*P*_0__ = *J*′(*v*_0_) − *L*′(*v*_0_) > 0, and (∂*D*/∂*w*)|_*P*_0__ = *K*′(*w*_0_) − *N*′(*w*_0_). So (∂*E*/∂*u*)|_(*P*_0_(*ɛ*),*ɛ*)_ = (∂*D*/∂*u*)|_*P*_0__ + *O*(*ɛ*) < 0, (∂*E*/∂*v*)|_(*P*_0_(*ɛ*),*ɛ*)_ = (∂*D*/∂*v*)|_*P*_0__ + *O*(*ɛ*) > 0, and (∂*E*/∂*w*)|_(*P*_0_(*ɛ*),*ɛ*)_ = (∂*D*/∂*w*)|_*P*_0__ + *O*(*ɛ*).

From Lemma 4, we get grad Δ|_(*P*_0_(*ɛ*),*ɛ*)_ = *k*((∂*D*/∂*u*)|_*P*_0__ + *O*(*ɛ*), (∂*D*/∂*v*)|_*P*_0__ + *O*(*ɛ*), (∂*D*/∂*w*)|_*P*_0__ + *O*(*ɛ*)).

From (19), we get that

(22) $$\begin{array}{c} \frac{dv}{du}=\frac{{\bar{I}}^{\prime }\left(u,ɛ\right)}{{\bar{J}}^{\prime }\left(v,ɛ\right)}e^{\left(\bar{I}(u,ɛ)-\bar{J}(v,ɛ)\right)/ɛ}>0,\\ \frac{dw}{du}=\frac{{\bar{M}}^{\prime }\left(u,ɛ\right)}{{\bar{N}}^{\prime }\left(w,ɛ\right)}e^{\left(\bar{M}(u,ɛ)-\bar{N}(w,ɛ)\right)/ɛ}>0. \end{array}$$

Noticing that *I*(*u*_0_) − *J*(*v*_0_) > 0, *M*(*u*_0_) − *N*(*w*_0_) = 0, we get that $e^{\left(\bar{I}(u,ɛ)-\bar{J}(v,ɛ)\right)/ɛ}\to +\infty$ , *ɛ* → 0^+^ and $e^{\left(\bar{M}(u,ɛ)-\bar{N}(w,ɛ)\right)/ɛ}=O(1)$ , *ɛ* → 0^+^.

Then for *ɛ* > 0 and small enough we get that

(23) dΔdu|(P0(ɛ),ɛ)=(∂Δ∂u+∂Δ∂vdvdu+∂Δ∂wdwdu)|(P0(ɛ),ɛ)=grad Δ|(P0(ɛ),ɛ)·(1,dvdu,dwdu)|(P0(ɛ),ɛ)≠0.

Because *ϕ*_*w*_ *φ*_*v*_ ≠ 0, $\left(d^{2}\bar{b}/du^{2}\right){|}_{\left(P_{0}(ɛ),ɛ)\right)}\neq 0$ is equivalent to (*d*Δ/*du*)|_(*P*_0_(*ɛ*),*ɛ*))_ ≠ 0. So we get that (*u*_0_(*ɛ*), *v*_0_(*ɛ*), *w*_0_(*ɛ*)) is a fold point of map Φ_*ɛ*_. So from Lemma 3 and fold bifurcation of scalar map $\bar{b}=\bar{\psi }(u,a,\bar{c},ɛ)$ (see [16]), we get that system (7) has two limit cycles near Γ_*uvw*_ by unfolding the parameters *a*, *b*, *c*.

The conclusion of the second part of Theorem 1 follows.

### 2.3. The Proof of the Third Part of Theorem 1

In this subsection, we give the proof of the third part of Theorem 1. First, we present the following lemma.

**Lemma 5**

As *ɛ* > 0 and small enough, if there exists ${\hat{P}}_{0}(ɛ)=(u_{0}(ɛ),v_{0}(u_{0},ɛ),w_{0}(u_{0},v_{0},ɛ))$ that satisfies Δ(*u*, *v*, *w*, *ɛ*) = 0, *d*Δ(*u*, *v*, *w*, *ɛ*)/*du* = 0, and $e^{(\bar{I}-\bar{J})/ɛ}=e^{(\bar{M}-\bar{N})/ɛ}$ , then it holds that

(24) $$\begin{array}{c} grad{\left(\frac{d\Delta }{du}\right)|}_{{\hat{P}}_{0}(ɛ)}=\hat{k}\cdot grad\;\hat{E}{|}_{{\hat{P}}_{0}(ɛ)}, \end{array}$$

where $\hat{E}(u,v,w,ɛ)=\bar{I}(u,ɛ)-\bar{J}(v,ɛ)-ɛ\ln(-{\bar{J}}^{\prime }{\bar{N}}^{\prime }\left(\partial D/\partial u\right)/\left({\bar{I}}^{\prime }{\bar{N}}^{\prime }\left(\partial D/\partial v\right)+{\bar{J}}^{\prime }{\bar{M}}^{\prime }\left(\partial D/\partial w\right)\right)+O(ɛ))$ , $grad\;\hat{E}=(\partial \hat{E}/\partial u,\partial \hat{E}/\partial v,\partial \hat{E}/\partial w)$ is the gradient of function $\hat{E}(u,v,w,ɛ)$ , and $\hat{k}$ is nonzero function at $\left({\hat{P}}_{0}(ɛ),ɛ\right)$ .

**Proof**

From the proof of the second part of Theorem 1, as Δ(*u*, *v*, *w*, *ɛ*) = 0 we get

(25) dΔdu=k[I¯′J¯′e(I¯−J¯)ɛ(∂D∂v+O(ɛ))+M¯′N¯′e(M¯−N¯)/ɛ(∂D∂w+O(ɛ))+(∂D∂u+O(ɛ))].

From $e^{(\bar{I}-\bar{J})/ɛ}=e^{(\bar{M}-\bar{N})/ɛ}$ , we get that *d*Δ/*du* = 0 is equivalent to

(26) $$\begin{array}{c} e^{(\bar{I}-\bar{J})/ɛ}=\frac{-\left(\partial D/\partial u\right){\bar{J}}^{\prime }{\bar{N}}^{\prime }}{{\bar{I}}^{\prime }{\bar{N}}^{\prime }\left(\partial D/\partial v\right)+{\bar{J}}^{\prime }{\bar{M}}^{\prime }\left(\partial D/\partial w\right)}+O\left(ɛ\right). \end{array}$$

So rewrite (26), and we get that the equation *d*Δ/*du* = 0 is equivalent to $\hat{E}(u,v,w,ɛ)=0$ under assumptions Δ = 0, $e^{(\bar{I}-\bar{J})/ɛ}=e^{(\bar{M}-\bar{N})/ɛ}$ for *ɛ* > 0 and small. That means that both equations *d*Δ(*u*, *v*, *w*, *ɛ*)/*du* = 0 and $\hat{E}(u,v,w,ɛ)=0$ determine the same surface $\hat{S}$ in the neighborhood of ${\hat{P}}_{0}(ɛ)$ . For $\text{grad}\;\left(d\Delta /du\right){|}_{{\hat{P}}_{0}(ɛ)}$ , $\text{grad}\;\hat{E}{|}_{{\hat{P}}_{0}(ɛ)}$ are both the normal vectors of surface $\hat{S}$ at the point ${\hat{P}}_{0}(ɛ)$ , then we get (24).

The proof of Lemma 5 is completed.

*Proof of the Third Part of Theorem 1.* From Lemmas 4 and 5, we get that

(27) $$\begin{array}{c} \Delta \left(u,v,w,ɛ\right)=0,\\ \frac{d\Delta \left(u,v,w,ɛ\right)}{du}=0 \end{array}$$

is equivalent to

(28) $$\begin{array}{c} E\left(u,v,w,ɛ\right)=0,\\ \hat{E}\left(u,v,w,ɛ\right)=0. \end{array}$$

It is easy to check that *E*(*u*_0_, *v*_0_, *w*_0_, 0) = 0, $\hat{E}(u_{0},v_{0},w_{0},0)=0$ and surfaces*S*_1_ : *E*(*u*, *v*, *w*, *ɛ*) = 0 and $S_{2}:\hat{E}(u,v,w,ɛ)=0$ transversely intersect along a curve *l*_*ɛ*_ which passes through point (*u*_0_, *v*_0_, *w*_0_, 0). Therefore, for *ɛ* > 0 and small enough, we choose point ${\hat{P}}_{0}(ɛ)=(u_{0}(ɛ),v_{0}(u_{0},ɛ),w_{0}(u_{0},v_{0},ɛ))$ on the curve *l*_*ɛ*_. Then $\Delta ({\hat{P}}_{0}(ɛ),ɛ)=0$ , $\left(d\Delta /du\right){|}_{\left({\hat{P}}_{0}(ɛ),ɛ\right)}=0$ .

Consider the map Φ_*ɛ*_ given in (19). By applying a similar process to the one in the proof the second part of Theorem 1, we get $\bar{b}=\bar{\psi }(u,a,c,ɛ)$ , $d\bar{b}/du=-\Delta /\phi_{w}\varphi_{v}$ , and $\left(d^{2}\bar{b}/du^{2}\right){|}_{\Delta =0}=-\left(d\Delta /du\right)/\phi_{w}\varphi_{v}$ . Next, under the assumptions given in the third part of Theorem 1, we compute the third derivative of $\bar{b}$ with respect to *u* at ${\hat{P}}_{0}(ɛ)$ and get

(29) d3b¯du3|P^0(ɛ)=−(d/du)(dΔ/du)ϕwφv|P^0(ɛ)=−1ϕwφv·grad(dΔdu)|P^0(ɛ)·(1,dvdu,dwdu)P^0(ɛ).

From Lemma 5, we get $\text{grad}\left(d\Delta /du\right){|}_{{\hat{P}}_{0}(ɛ)}=\hat{k}\cdot (I^{\prime }(u_{0})+O(ɛ),J^{\prime }(v_{0})+O(ɛ),O(ɛ))$ .

From (19) and (26), we get that

(30) dvdu|P^0(ɛ)=[I¯′(u,ɛ)J¯′(v,ɛ)e(I¯(u,ɛ)−J¯(v,ɛ))/ɛ]P^0(ɛ)=[−I′N′(∂D/∂u)I′N′(∂D/∂v)+J′M′(∂D/∂w)]P0+O(ɛ),dwdu|P^0(ɛ)=[M¯′(u,ɛ)N¯′(w,ɛ)e(M¯(u,ɛ)−N¯(w,ɛ))/ɛ]P^0(ɛ)=[−J′M′(∂D/∂u)I′N′(∂D/∂v)+J′M′(∂D/∂w)]P0+O(ɛ).

Then for *ɛ* > 0 and small enough we get that

(31) d2Δdu2|P^0(ɛ) =k^·(I′(u0)+J′(v0)×[−I′N′(∂D/∂u)I′N′(∂D/∂v)+J′M′(∂D/∂w)]P0+O(ɛ)) =I′(u0)k^·[J′K′M′−I′L′N′I′N′(∂D/∂v)+J′M′(∂D/∂w)+O(ɛ)]P0 ≠0.

For $\left(\hat{k}/\phi_{w}\varphi_{v}\right){|}_{{\hat{P}}_{0}(ɛ)}\neq 0$ , $\left(d^{3}\bar{b}/du^{3}\right){|}_{{\hat{P}}_{0}(ɛ)}\neq 0$ is equivalent to $\left(d^{2}\Delta /du^{2}\right){|}_{{\hat{P}}_{0}(ɛ)}\neq 0$ ; then we get that ${\hat{P}}_{0}(ɛ)$ is a cusp point of map $\bar{b}=\bar{\psi }(u,a,\bar{c},ɛ)$ for *ɛ* > 0 and small enough. From Lemma 3 and cusp bifurcation of scalar map [16], we get that the above degenerated limit cycle is generically unfolded by the parameters (*a*, *b*, *c*), for *ɛ* > 0 and small enough, producing system (7) having three hyperbolic limit cycles near Γ_*uvw*_.

The proof of the third part of Theorem 1 is completed.

## 3. Application to Polynomial Liénard Equation

In this section, we will apply Theorem 1 to the following polynomial Liénard equation:

(32) $$\begin{array}{c} X_{a,b,c,ɛ}:\{\begin{array}{cc} \dot{x}=y-\left(-\frac{1}{6}x^{6}+\frac{5}{4}x^{4}-2x^{2}+ax\right), & \\ \dot{y}=ɛ(b-\left(x-1\right) & \\ \times \left(1+c_{1}\left(x-1\right)+c_{2}{\left(x-1\right)}^{3}\right)) & \\ \;\;\times \left(c-\left(x+1\right)\right)\left(-x\right). & \end{array} \end{array}$$

For this system, *F*_*a*_(*x*) = (−(1/6)*x* ^6^ + (5/4)*x* ^4^ − 2*x* ^2^ + *ax*). Here we use symbols *p*_*i*_, *i* = 1,2, 3,4 defined in Section 2. By direct computation we get that *p*_1_ = −2, *p*_4_ = 2; the values of *F*_0_(*x*) at these two points are both 4/3 and the values of *F*_0_(*x*) at the points *p*_2_ = −1, *p*_3_ = 1 are both −11/12 and *F*_0_(0) = 0.

**Figure 5:**
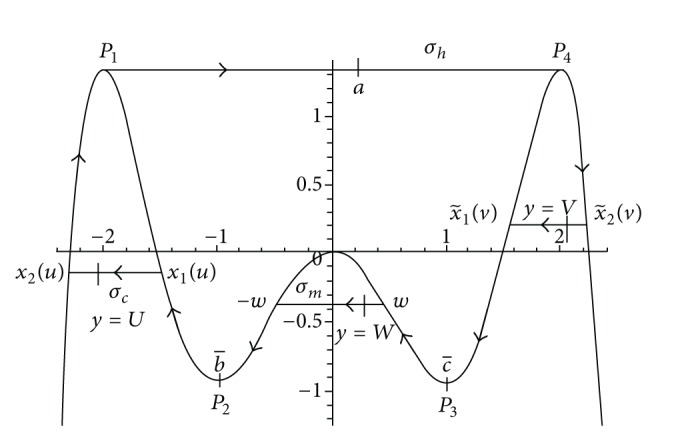
Canard cycles Γ_*UVW*_ of system (32).

For a given *V* ∈ (−11/12, 0), the equation *F*_0_(*x*) = *V* will have three roots on the right side of the *y*-axis. For the roots, the formula in the above equation contains complex number, so we choose the smallest one of the above three roots and denote it by the variable *v*, where *v* ∈ (0,1). By noting that relation between *u* and *v* is one to one relation, we express the other two roots in the form ${\tilde{x}}_{1}(v)=\left(1/2\right)(\sqrt{15-2v^{2}-\sqrt{3}\sqrt{11+20v^{2}-4v^{4}}})$ , ${\tilde{x}}_{2}(v)=\left(1/2\right)(\sqrt{15-2v^{2}+\sqrt{3}\sqrt{11+20v^{2}-4v^{4}}})$ , where $v<{\tilde{x}}_{1}(v)<{\tilde{x}}_{2}(v)$ , *v* ∈ (0,1). Using the same way, we take *y* = *U*, *U* ∈ (−11/12,0); then the equation *F*_0_(*x*) = *U* will have three roots on the left side of the *y*-axis; we choose the biggest one of the above three roots and denote it by the variable *u*; we express the other two roots in the form $x_{1}(u)=-{\tilde{x}}_{1}(u)$ , $x_{2}(u)=-{\tilde{x}}_{2}(u)$ and *x*_2_(*u*) < *x*_1_(*u*) < *u*, *u* ∈ (−1,0). Also *F*_0_(*x*) = *y* is symmetrical about 0, so the straight line *y* = *W*, *W* ∈ (−11/12, 0), between −1 and 1 will intersect the curve with two points; then we, respectively, denote their *x*-coordinate by −*w*, *w*, where *w* ∈ (0,1). The corresponding canard cycle Γ_*UVW*_ can be seen in Figure 5.

Let *P*(*x*) = (1/8)*x* ^8^ − (3/2)*x* ^6^ + 6*x* ^4^ − 8*x* ^2^, *Q*_1_(*x*) = −(1/9)*x* ^9^ + (1/8)*x* ^8^ + (9/7)*x* ^7^ − (24/5)*x* ^5^ − (3/2)*x* ^6^ + 6*x* ^4^ + (16/3)*x* ^3^ − 8*x* ^2^, *Q*_2_(*x*) = −(1/11)*x* ^11^ + (3/10)*x* ^10^ + (2/3)*x* ^9^ − (13/4)*x* ^8^ + (3/7)*x* ^7^ + (21/2)*x* ^6^ − (56/5)*x* ^5^ − 6*x* ^4^ + 16*x* ^3^ − 8*x* ^2^; then from the slow divergence integral formula (8) in Section 2, we get that

(33) M(u)=P(−1)−P(−x~1(u)) +c1(Q1(−1)−Q1(−x~1(u))) +c2(Q2(−1)−Q2(−x~1(u))) +O((|c1|+|c2|)2),N(w)=P(−1)−P(−w) +c1(Q1(−1)−Q1(−w)) +c2(Q2(−1)−Q2(−w)) +O((|c1|+|c2|)2),I(u)=P(−2)−P(−x~2(u)) +c1(Q1(−2)−Q1(−x~2(u))) +c2(Q2(−2)−Q2(−x~2(u))) +O((|c1|+|c2|)2),J(v)=P(2)−P(x~2(v)) +c1(Q1(2)−Q1(x~2(v))) +c2(Q2(2)−Q2(x~2(v))) +O((|c1|+|c2|)2),L(v)=P(1)−P(x~1(v)) +c1(Q1(1)−Q1(x~1(v))) +c2(Q2(1)−Q2(x~1(v))) +O((|c1|+|c2|)2),K(w)=P(1)−P(w)+c1(Q1(1)−Q1(w)) +c2(Q2(1)−Q2(w)) +O((|c1|+|c2|)2).

Firstly, consider *M*(*u*) − *N*(*w*) = 0; that is,

(34) P(−w)−P(−x~1(u))+c1(Q1(−w)−Q1(−x~1(u))) +c2(Q2(−w)−Q2(−x~1(u)))+O((|c1|+|c2|)2)=0.

When *c*_1_ = *c*_2_ = 0, from the fact that the function *P*(*x*) is monotone on the interval (−2, −1) we get $w={\tilde{x}}_{1}(u)$ . Let $w={\tilde{x}}_{1}(u)+c_{1}w_{1}(u)+c_{2}w_{2}(u)+O({\left(|c_{1}|+|c_{2}|\right)}^{2})$ and substitute it into (34); by setting the coefficients of *c*_1_ and *c*_2_ of obtained equation to equal zeros, we get *w*_1_(*u*) = 0, *w*_2_(*u*) = 0. Therefore, we get

(35) $$\begin{array}{c} w\left(u\right)={\tilde{x}}_{1}\left(u\right)+O\left({\left(\left|c_{1}\right|+\left|c_{2}\right|\right)}^{2}\right). \end{array}$$

Secondly, consider *I*(*u*) − *J*(*v*) = 0; that is,

(36) P(x~2(v))−P(−x~2(u)) +c1(Q1(−2))−Q1(2)+Q1(x~2(v)) −Q1(−x~2(u))+c2(Q2(−2))−Q2(2)+Q2(x~2(v)) −Q2(−x~2(u))+O((|c1|+|c2|)2)=0.

By noticing that the even function *P*(*x*) is strictly monotone on the interval (−2, −1) and ${\tilde{x}}_{2}(-u)={\tilde{x}}_{2}(u)$ , we get *v* = −*u* as *c*_1_ = *c*_2_ = 0.

Let

(37) $$\begin{array}{c} v=-u+c_{1}v_{1}\left(u\right)+c_{2}v_{2}\left(u\right)+O\left({\left(\left|c_{1}\right|+\left|c_{2}\right|\right)}^{2}\right), \end{array}$$

and put it into (36); then by setting the coefficients of *c*_1_ and *c*_2_ of obtained equation to equal zeros, we get

(38) $$\begin{array}{c} v_{1}\left(u\right)=\frac{Q_{1}\left(2\right)-Q_{1}\left(-2\right)+Q_{1}\left(-{\tilde{x}}_{2}\left(u\right)\right)-Q_{1}\left({\tilde{x}}_{2}\left(u\right)\right)}{P^{\prime }\left({\tilde{x}}_{2}\left(-u\right)\right){\tilde{x}}_{2}^{\prime }\left(-u\right)},\\ v_{2}\left(u\right)=\frac{Q_{2}\left(2\right)-Q_{2}\left(-2\right)+Q_{2}\left(-{\tilde{x}}_{2}\left(u\right)\right)-Q_{2}\left({\tilde{x}}_{2}\left(u\right)\right)}{P^{\prime }\left({\tilde{x}}_{2}\left(-u\right)\right){\tilde{x}}_{2}^{\prime }\left(-u\right)}. \end{array}$$

Thirdly, consider function *L*(*v*) − *K*(*w*); that is,

(39) L(v)−K(w)=P(w)−P(x~1(v))+c1(Q1(w)) −Q1(x~1(v))+c2(Q2(w)−Q2(x~1(v))) +O((|c1|+|c2|)2).

Putting the expressions (35) and (37) into (39), we get

(40) $$\begin{array}{c} L\left(v\right)-K\left(w\right)=c_{1}\zeta_{1}\left(u\right)+c_{2}\zeta_{2}\left(u\right)+O{\left(\left|c_{1}\right|+\left|c_{2}\right|\right)}^{2}, \end{array}$$

where

(41) ζ1(u)=−P′(x~1(−u))x~1′(−u) ×Q1(2)−Q1(−2)+Q1(−x~2(u))−Q1(x~2(u))P′(x~2(−u))x~2′(−u),ζ2(u)=−P′(x~1(−u))x~1′(−u) ×Q2(2)−Q2(−2)+Q2(−x~2(u))−Q2(x~2(u))P′(x~2(−u))x~2′(−u).

From the definition of function *Q*_*i*_(*x*), *i* = 1,2, we get that *ζ*_1_(*u*) · *ζ*_2_(*u*) ≠ 0, *u* ∈ (−1,0).

Denote $\eta_{1}(u)=Q_{1}(2)-Q_{1}(-2)+Q_{1}(-{\tilde{x}}_{2}(u))-Q_{1}({\tilde{x}}_{2}(u))$ , $\eta_{2}(u)=Q_{2}(2)-Q_{2}(-2)+Q_{2}(-{\tilde{x}}_{2}(u))-$ $Q_{2}({\tilde{x}}_{2}(u))$ and denote

(42) $$\begin{array}{c} \theta \left(u\right)=-\frac{P^{\prime }\left({\tilde{x}}_{1}\left(-u\right)\right){\tilde{x}}_{1}^{\prime }\left(-u\right)}{P^{\prime }\left({\tilde{x}}_{2}\left(-u\right)\right){\tilde{x}}_{2}^{\prime }\left(-u\right)}. \end{array}$$

For *θ*(*u*) ≠ 0, *u* ∈ (−1,0), then *ζ*_1_(*u*)/*ζ*_2_(*u*) = *η*_1_(*u*)/*η*_2_(*u*).

Denote *H*(*u*) = *L*(*v*(*u*)) − *K*(*w*(*u*)), where *w*(*u*) and *v*(*u*) are, respectively, implicitly determined by *M*(*u*) − *N*(*w*) = 0 and *I*(*u*) − *J*(*v*) = 0.

By direct computations, we get

(43) dH(u)du=L′·v′(u)−K′·w′(u)=L′·I′J′−K′·M′N′=I′L′N′−J′K′M′J′N′.

On the other hand from expressions (40), we get that

(44) $$\begin{array}{c} \frac{dH\left(u\right)}{du}=c_{1}\zeta_{1}^{\prime }\left(u\right)+c_{2}\zeta_{2}^{\prime }\left(u\right)+O{\left(\left|c_{1}\right|+\left|c_{2}\right|\right)}^{2}. \end{array}$$

As *H*(*u*) = 0, then we get

(45) $$\begin{array}{c} \frac{dH\left(u\right)}{du}=c_{1}\theta \left(u\right)\eta_{1}^{\prime }\left(u\right)+c_{2}\theta \left(u\right)\eta_{2}^{\prime }\left(u\right)+O{\left(\left|c_{1}\right|+\left|c_{2}\right|\right)}^{2}. \end{array}$$

**Figure 6:**
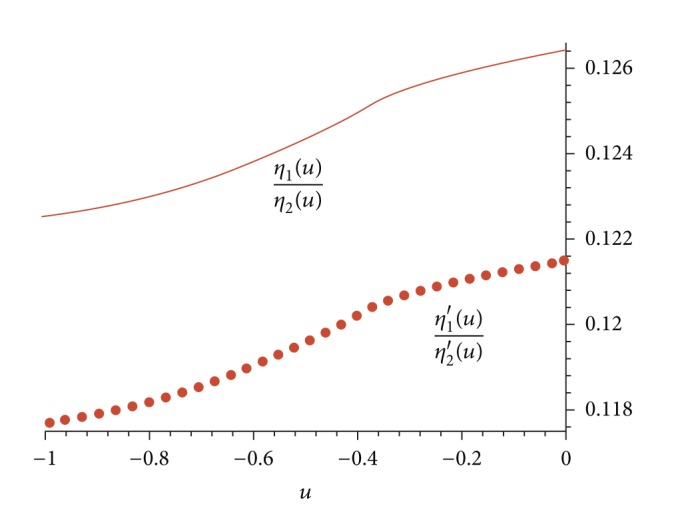
The graph of functions *η*_1_(*u*)/*η*_2_(*u*) and *η*_1_′(*u*)/*η*_2_′(*u*) on the interval (−1,0) are, respectively, plotted in solid line and point line.

The graph of functions *η*_1_(*u*)/*η*_2_(*u*) and *η*_1_′(*u*)/*η*_2_′(*u*) on the interval (−1,0) is plotted in Figure 6.

From the graph in Figure 6, we conclude that, for any *u*_0_ ∈ (−1,0), there exists *c*_2_ = −(*η*_2_(*u*_0_)/*η*_1_(*u*_0_))*c*_1_ + *O*(*c*_1_^2^), 0 < |*c*_1_ | ≪1 such that equation *H*(*u*) = 0 holds and *dH*(*u*_0_)/*du* = *c*_1_ *θ*(*u*_0_)(*η*_1_′(*u*_0_) + (*η*_1_(*u*_0_)/*η*_2_(*u*_0_))*η*_2_′(*u*_0_)) + *O*(*c*_1_^2^) ≠ 0 for *c*_1_ > 0 and small enough.

In other words, as *c*_2_ = −(*η*_2_(*u*_0_)/*η*_1_(*u*_0_))*c*_1_ + *O*(*c*_1_^2^), 0 < |*c*_1_ | ≪1, it holds that *I*′(*u*_0_)*L*′(*v*_0_)*N*′(*w*_0_) − *J*′(*v*_0_)*K*′(*w*_0_)*M*′(*u*_0_) ≠ 0, where *w*_0_ = *w*(*u*_0_), *v*_0_ = *v*(*u*_0_). So there exists (*u*_0_, *v*_0_, *w*_0_) which satisfies the conditions in the third part of Theorem 1, and from conclusions of Theorem 1, we get that system (32) has three hyperbolic relaxation oscillations near canard cycle Γ_*UVW*_, where *U* = *F*_0_(*u*_0_), *V* = *F*_0_(*v*(*u*_0_)), *W* = *F*_0_(*w*(*u*_0_)), *u*_0_ ∈ (−1,0).
